# Track Differences in Civic and Democratic Engagement During Secondary Education: A New Panel Study From the Netherlands

**DOI:** 10.1111/1468-4446.70006

**Published:** 2025-06-30

**Authors:** Herman van de Werfhorst, Geert ten Dam, Sara Geven, Twan Huijsmans, Hester Mennes, Laura Mulder, Jaap van Slageren, Tom van der Meer

**Affiliations:** ^1^ European University Institute Florence Italy; ^2^ University of Amsterdam Amsterdam the Netherlands; ^3^ Utrecht University Utrecht the Netherlands

## Abstract

Whether students educated in different ability tracks in secondary education develop different levels of civic and democratic engagement is yet unclear. To explore this issue, we focus on how schools bring students of different tracks and family backgrounds together, and whether such between‐school differences are associated with varying growth rates in civic and democratic engagement during secondary education. Using newly collected 4‐year panel data starting at the very beginning of the Dutch tracked educational system, the Dutch Adolescent Panel on Democratic Values (DAPDV), we study developments in institutional trust, societal interest, voting intention, and political knowledge. Growth curve models show that much of the variation between tracks and between schools is rather stable, although track differences in institutional trust became more pronounced. Although schools that are more compositionally diverse vary from homogeneous schools, track differences are largely present already at the start of secondary education. Within‐individual transition models show that students moving up to more advanced tracks do gain in political knowledge.

## 1 Introduction

Horizontal differences within levels of educational attainment have received prominence in social science research. Inequalities in and through education are not only produced in different levels of education (e.g., college vs. high school), but also within levels of attainment. One important source of stratification concerns the different tracks in secondary school (e.g., pre‐vocational vs. academic tracks) (Lucas 2001; Oakes 2005). Early tracking is associated with larger inequalities in educational achievement and attainment by socioeconomic background and lower intergenerational social mobility (Terrin and Triventi 2022; Strello et al. 2021; Reichelt et al. 2019). Students of disadvantaged backgrounds tend to be overrepresented in the vocational trajectories, and middle‐class children in the academic trajectories, because of differences in academic achievement and difference in choices on top of achievement (Kloosterman et al. 2009). Moreover, a relatively new discovery is that students educated in different tracks also vary with regard to civic and political engagement, and that early tracking systems seem to magnify gaps in engagement compared to comprehensive systems (Hoskins et al. 2014; Janmaat and Mons 2011; Van de Werfhorst 2007, 2017; Witschge and Van de Werfhorst 2020). Earlier it had already been demonstrated how tracks differ in the type of civic education that is offered, although without much focus on the impacts on engagement (Niemi and Junn 1998; G. T. M. Ten Dam and Volman 2003). This suggests that educational tracks in secondary education directly influence students' civic engagement.

Nevertheless, studies on developments in civic and democratic engagement over the school career are mixed, causing debates about whether track effects result from selection or causation. Sometimes (some) growing track differences in engagement during the school career are found, suggestive of causal effects of tracking (Eckstein et al. 2012; Hoskins and Janmaat 2016; Witschge et al. 2019). Others find no growing gaps, and instead find that track differences already existed before students entered different tracks (Persson 2012). It is fair to say that the jury is still out on whether track differences result from the track enrolled, or from selection processes into tracks, and on the question through which mechanisms such causal or selection effects are manifested. It is, however, crucial to understand differentiations in the formation of civic and democratic engagement among the younger generation. Education has historically been seen as relevant for engagement with society, guiding students into the adoption of its dominant norms (Durkheim 1922) and in particular socialising toward democratic engagement (Dewey 1966). Potential differences in civic and democratic engagement between education groups may, then, illustrate democratic inequalities that can be considered unfair in light of the relationship between the state and its citizens (Miller 1999). And besides such a “vertical” relationship, a Durkheimian view on education implies that “horizontal” engagement with fellow citizens is equally crucial for social order and cohesion to be maintained. The study of the development of civic and democratic engagement is therefore crucial to assess an important task of education. Moreover, such a study can help us to understand what the potential sources of inequalities in civic and democratic engagement may look like (Hooghe 2004; McFarland and Thomas 2006).

One reason why the evidence is mixed is that track differences (and their development over time) may depend on the school context, and in particular on how students of different tracks are brought together. Some schools offer multiple tracks, while other schools offer only one, or a subset of them. Also, schools vary in the extent to which they integrate students of various socioeconomic backgrounds. Track differences and their growth can depend on the school context in opposing ways. On the one hand, if different groups are brought together in one school, differences between tracks may become more visible, thereby potentially undermining the self‐efficacy and motivation of students in the lowest ability tracks, also in the domain of politics (Janmaat and Mons 2023). Sociodemographically diverse classes may be also ideologically diverse, making it harder for schools to offer an open classroom climate (Knowles 2021). Research on track differences in study orientations shows that, similarly, differences between tracks may become more visible in broader schools, leading to larger gaps between tracks (Van Houtte and Stevens 2009).

On the other hand, integrated schools may also enhance social interactions between groups, leading to better understanding of each other, and a growing concern with society‐at‐large. The research questions guiding our investigation are: What is the development in terms of civic and democratic engagement in different school tracks during the secondary school career? Does the (changing) difference between tracks depend on how various students are brought together in the school?

We use newly collected panel data from the Netherlands, the Dutch Adolescent Panel on Democratic Values (DAPDV), to understand the role of schools for the development of democratic orientation between grade 7 and 10 in secondary schools. Our study advances upon previous research in the following ways. First, we study how tracking effects may be modified by the tracking structure. Second, we study the development of civic and democratic engagement across tracks right from the moment that tracking starts, while other studies often look at later tendencies (e.g., Hoskins and Janmaat 2016; Witschge and Van de Werfhorst 2020). Third, we study a range of indicators of civic and democratic engagement, both of a political and general societal kind.

## 2 Theoretical Background

### 2.1 Tracking and Political Socialisation

To understand the role of tracks and schools for the formation of civic and democratic engagement, it is helpful to integrate various theoretical perspectives on political socialisation, inequality in political engagement, contact theory, and educational differentiation and polarisation. Importantly, the high school years are a crucial phase to study the development of civic and democratic engagement, as change is happening during that period (Geboers et al. 2013).

Starting with political socialisation theory, important agents for political socialisation are the school and the family. The school is potentially important for political and civic socialisation, especially when schools have an open and democratic classroom climate, a formalised citizenship and civic education curriculum, formal teacher civic training, and a range of democratic activities both inside and outside the school (Geboers et al. 2013; Persson 2015a; Wiseman et al. 2011; Mennes et al. 2023). Panel studies focusing on growth showed that civic education is associated with civic outcomes (Dassonneville et al. 2012; Neundorf et al. 2016). Importantly, nationally centralised policies on civic education are associated with reduced dispersions in civic engagement among the student body in society (Witschge and Van de Werfhorst 2016). Tracks differ in the kinds of civic skills that are addressed, with academic tracks being more oriented to critical skills relevant for wider societal participation, and vocational tracks more toward the acquisition of social skills that enhance the opportunities of students themselves (G. T. M. Ten Dam and Volman 2003). A school‐level study furthermore showed that academic performance and civic skills of students are positively correlated, especially in the academic tracks (Mennes, van de Werfhorst, et al. 2023). Hence, it may be that students in academic tracks also show faster growth rates in civic and democratic engagement. Students in academic tracks are better able to see complexities in competing democratic principles (Thijs et al. 2024). Together, these tendencies could magnify democratic inequalities, which are ultimately detrimental for the level of social cohesion in society (Dewey 1966). Through education, societal norms can be transmitted, but if this happens differently depending on a student's educational context, varieties emerge that could harm vertical (i.e., between the state and its citizens) and horizontal (between citizens) patterns of social cohesion (Durkheim 1922; Dewey 1966; Barrett and Zani 2015).

While the school is thus often portrayed as an important environment for political and democratic socialisation, parents are arguably even more important. As early as at the start of primary school, there are social gradients in democratic understanding (Van Deth et al. 2011). In adolescence, political polarisation seems to be predicted by parents (Tyler and Iyengar 2023). Parents are particularly relevant agents of political socialisation depending on their own engagement and parenting style (Dinas 2014; Murray and Mulvaney 2012). Even if our focus is on schools in the current study, parents are relevant, as the selection of students into ability tracks is contingent on family background.

Much of the education‐oriented research focuses on educational processes that may enhance civic and democratic engagement, such as the classes that are taught or the quality of instruction. Following the terminology of the civic engagement literature, such educational processes affect the “resources” that students have at their disposal. However, civic engagement is not only promoted by resources but also by “recruitment networks” that may also vary across tracks and schools (Brady et al. 1995). Schools differ in the kinds of students that are encountered, in terms of their own academic, civic and parental background. In vocational tracks, students may encounter fewer students with skills and backgrounds conducive to engagement than in academic tracks. Moreover, besides resources and networks, differentiated schooling systems may also create (self‐) identification of students as members of a particular social class. Welfare state theorists have argued that stratified educational systems emphasise within‐group homogeneity and between‐group differences (Marshall 1950). A cultural sociological approach emphasises, in addition, the preformative functioning of education types, creating categories that “become real” and get cultural meaning (Domina et al. 2017; Lamont et al. 2014). Stereotypes about politics being a field for the well‐educated, or at a great distance of the working classes, may become salient during secondary education. Students in specific tracks identify with their educational group, which could enhance polarisation in the outcomes that schools aim to foster, such as civic engagement (Van Houtte 2006; Knowles 2021). Thus, the practice of differentiating students at a life phase when the groundwork is laid for the formation of engagement may create stronger differences between groups in the participation in, and engagement with, society‐at‐large (cf. Van de Werfhorst 2017). As the *“tracking hypothesis”,* we can then predict that students in the academic track are more strongly engaged with democratic and societal institutions than students in the pre‐vocational track, and that this gap increases over the school grades.

### 2.2 Bringing Students Together: Opposing Forces?

The question now is how the school context may matter for how strongly stratifying track is for civic and democratic engagement. Given the myriad of different findings on track effects on engagement, our main focus is on how the school context may matter for the existence of track differences and their dynamic pattern. We are particularly interested in the question whether schools that bring together students from different tracks and backgrounds have different patterns of associations between the track an individual student is enrolled in, and the level of civic and democratic engagement displayed. There are two opposing views regarding the integrative function of schools that are inclusive with regard to the various tracks on offer and the backgrounds that the schools includes.

The first argument departs from intergroup contact theory. Intergroup contact theory stipulates that, under certain conditions, more intergroup contact reduces prejudice against other groups (Allport 1979[1954]; Pettigrew et al. 2011). Intergroup contact may not only reduce prejudice, but may also enable more social interactions with other groups, leading to more permeable boundaries between education groups in terms of the resources and networks that enable political and civic participation and engagement. Thus, a setting that brings various students together can promote intergroup contact, reduce prejudices, and minimise the self‐identification as a “category” in the educational system. Students of various tracks or socioeconomic backgrounds come together in more broadly composed schools, which may stimulate students to see society as a collective responsibility, leading to smaller gaps between tracks in the support for democracy, the level of trust in institutions, and the interest students have in societal issues. The *integration hypothesis* predicts that differences across tracks are *smaller* if schools bring various tracks and students of various socioeconomic backgrounds together.

However, an opposing argument would follow from works that connect the differentiation‐polarisation argument to the context of broader schools or comprehensive school settings. As Van Houtte and Stevens (2009) demonstrate for a system with a tracked educational system, cultural processes of self‐identification get magnified if schools integrate multiple tracks, leading to feelings of inferiority among students of the vocational track. Similarly, the Big Fish Little Pond effect—the pattern that, given one's own performance, students have lower levels of self‐efficacy in high‐performing schools than in low‐performing schools because they compare themselves with the school average—is shown to be correlated to the tracking of the schooling system (Parker et al. 2021). Lower‐achievers compare themselves with high‐performing students more in comprehensive schools than in tracked schools, and may feel more “futile” (Van Houtte and Stevens 2015). Such patterns can easily extend from self‐efficacy to civic and democratic engagement. Democracy and civic engagement require political self‐efficacy (Janmaat and Mons 2023), and the belief that democratic engagement is something more typical for students in the academic track may get stronger developed if students of various backgrounds are brought together. Thus, schools that integrate various tracks and socioeconomic backgrounds may enhance gaps between students in different tracks in civic and democratic engagement. One well‐known condition for contact theory to hold is that the groups must have an underlying equality (Pettigrew et al. 2011). In the absence of equality, as may be the case in a tracked educational system, intergroup contact is unlikely to diminish prejudices, and the cultural process of categorisation may get more prominent precisely if students of different tracks are confronted with each other. Students in the prevocational track may think that contributing to democracy is not for them. They may be less able to develop a connection to the broader society, when they are more evidently confronted with their comparatively disadvantaged position in society. The resulting *polarisation hypothesis* thus stipulates that differences across tracks are *larger* if schools bring various tracks and students of various socioeconomic backgrounds together.

Besides these arguments on the effects of schooling, an alternative thesis is that educational gaps in civic and democratic engagement are a consequence of selection into tracks. Education is, following this argument, a “proxy” for earlier life experiences (Kam and Palmer 2008; Persson 2015b), in our case most notably relating to the family background, or personal traits related to track enrolment (including intelligence or academic ability). Track differences in voting intention in France were for a large part attributable to the socioeconomic composition of schools (Janmaat and Mons 2023). A dynamic study of democratic and civic engagement can shed light on these issues as we can study growth in engagement on top of initial differences that may result from various forms of selection. The data can inform us whether selection takes place only in terms of the entry level of engagement, or that selection also happens regarding the trajectory during high school.

## 3 Research Design

### 3.1 The Dutch Adolescent Panel on Democratic Values

The development of civic and democratic engagement is still understudied in the political and social sciences, as the requirements are quite strong regarding the data. Valuable data meet at least three criteria. First of all, one needs panel data to track developments during the formative years. Second, to assess the relevance of different trajectories in education, one needs to start early in the school career, namely as early as trajectories start to vary. Third, one needs representative variation in the types of schools, preferably through random sampling of schools.

To meet these three criteria, we collected nationally representative panel data of secondary school students in the Netherlands, the Dutch Adolescent Panel on Democratic Values (DAPDV; Huijsmans et al. 2024). The data were collected between 2018 and 2024 in collaboration with schools. Secondary school starts at grade 7 in the Netherlands, when most students are 12 years old. The first round of data collection was carried out in October 2018, when students had just entered secondary education. This is the moment when the school system starts to become formally stratified. In particular, as the UNESCO report by Gromada et al. (2018) demonstrated, the Dutch educational system is highly equal in the primary schools (showing the lowest dispersion in academic performance of 38 advanced economies), but highly unequal in the secondary schools (ranked in the top third in terms of dispersion in academic performance). Hence, it is at the transition to secondary school when school careers really start to diverge.

Secondary schools were selected through a stratified sampling approach on the municipality level considering location and urbanity. All schools in the selected municipalities were contacted, which resulted in a sample of 49 schools with 210 seventh‐grade classes. The response rate at the level of schools was 35.8%. Due to the GDPR regulations we needed to obtain active consent from parents or caretakers, which was obtained for 46% of the students (82% of those whose consent form was returned). We excluded students in the “practical education” track (6% of our data), which is meant for those for whom the prevocational tracks are considered inadequate. Practical education focuses on students with IQ levels between 55 and 80, and with at least 3 years of delay in mathematics and literacy skills.

A total number of 2029 students participated in the first year of data collection in the autumn of 2018. Of these students, 1494 participated eventually in more than 1 year of data collection, which was a condition to be included in the present analyses.^1^ Of these, 600 took part in two waves, 487 in three waves, and 407 in all four waves. The Dutch secondary school system is characterised by early tracking into different educational levels, and these different tracks have different lengths. The tracks preparing for lower vocational education (vmbo) has a duration of 4 years, the track preparing for higher professional education (havo) takes 5 years, and the academic track preparing for university (vwo) takes 6 years. Since we want our sample to be balanced over time in terms of the educational level of the students, we only use the first four waves of the data when students of all tracks are still included in the panel.

The data are exceptionally rich with regard to the measurement of civic and democratic engagement, has run over 6 years, and covers many different schools. Altogether this is arguably the most elaborate school‐based panel study on civic and democratic engagement, and promises to be an important source for studying track differences.

### 3.2 Variables

Civic engagement is a multidimensional concept comprising of behaviours, values, attitudes and knowledge for contributing to community and a democratic society (Amnå 2012; Wray‐Lake and Shubert 2019). We study four variables measuring different aspects of civic and democratic engagement, all separately assessed per year, yielding time‐varying dependent variables. Together, these variables relate to both the vertical and horizontal patterns of social cohesion, with vertical patterns referring to the relationship between the (democratic) state and its inhabitants, and horizontal patterns to the relationships among inhabitants of a society. In both of these patterns, differences in engagement may be threatening to social cohesion in society, for instance when some groups are more likely to take part in politics or trust institutions (vertical), or some groups are more strongly interested in societal issues (horizontal). First, we study *institutional trust*, a well‐known indicator of social cohesion in society (Rothstein and Stolle 2008; Twenge et al. 2014). Our scale includes trust in judges, police officers, politicians, the army, and medical doctors. Cronbach alphas were generally high (between 0.79 and 0.82 across the years). We constructed a factor score per year. Second, we studied *societal interest*, by asking students to what extent they are interested in the topics of poverty, climate change, crime, politics, racism, terrorism, refugees. Cronbach alphas were generally high, between 0.73 and 0.82 across the years. We created a factor score per year. Then, we measured *voting intention* by asking whether students had the intention to go to vote after turn 18 (in four categories from certainly not to certainly yes). Lastly, we tested *political knowledge* with six items regarding factual knowledge on elections and the political legislative process (the sum of correct answers is the score on the variable) (this last variable was only available from the eighth grade onwards). Together, these variables are important indicators of democratic and civic competences, that foster engagement. They speak to well‐known theories of civic and citizenship engagement, and political engagement. The variables represent both social and political dimensions, in line with Barrett and Zani's conceptualisation of political and civic aspects of engagement. Similar to the citizenship education literature the set includes attitudinal, behavioural (or intentional) and knowledge‐based components of civic competences and engagement (Barrett and Zani 2015; G. Ten Dam et al. 2011, 2020).

*School track* is operationalised in three categories: pre‐vocational, mixed/intermediate, and academic. The pre‐vocational category includes students attending any of the four pre‐vocational tracks which prepare students for vocational school/apprenticeship. The mixed/intermediate category combines students (1) attending the intermediate track that prepares students for attending a university of applied sciences and (2) students in mixed track (integrating multiple tracks, always including the intermediate track). The academic track is the track that prepares for attending a research university. Given that, in some schools, 7th grade students are placed in mixed tracks (i.e., combinations of at least two of the three main tracks), while in other schools students enrol in one track (often offering only one track in single‐track school), we want to examine whether track differences are smaller when students are more often placed in mixed tracks. However, in later grades the mixed track no longer exists, as all students in ninth grade have chosen one of the three main tracks. By classifying tracks the way we do, we can study growth trajectories as a function of time‐varying track placements in various school settings, including, for instance, common trajectories from mixed tracks to either the pre‐vocational or academic track. Our focus is particularly on the pre‐vocational and academic tracks, as they are dynamically contrasted with the middle category.

*Socioeconomic background* is measured using factor scores on a factor of two variables, the number of books in the household, and a subjective indicator on family economic position (“how rich is your family in relation to other families?”, with a five‐point scale from “much less rich” to “much richer”). This variable is measured in a time‐constant way, by using the grade‐7 data and filling up the data with later grades in case of missing values. We follow a Mundlak‐type standardisation of socioeconomic status, by centring the z‐scored SES score at the level of school*grade, and add the mean SES score by school*grade (see below, Bell et al. 2019; Mundlak 1978). This means that possible track differences in mean or growth in civic and democratic engagement are controlled for selection into schools based on (measured) average socioeconomic status. To study the SES heterogeneity, we also use the standard deviation in the individual SES indicator by school*grade.

Our models control for *gender* (in a binary dummy, male = 0, female = 1) and migration background (no migration background, at least one parent born in an OECD country, or at least one parent born in a non‐OECD country).

Table 1 shows descriptive statistics on all variables, on the person‐grade file.

**TABLE 1:** Descriptive statistics.

Variable	Mean	SD	Range	*N*
Institutional trust	0.001	0.892	5.132	3965
Societal interest	0.003	0.875	4.751	4163
Voting intention	2.166	0.778	3	3942
Political knowledge	3.912	1.754	6	2739
Proportion in a mixed track in grade 7	0.362	0.345	0.971	4233
Socioeconomic status (centred by school‐grade)	0	0.857	6.177	3832
School‐grade mean SES (time‐varying)	−0.018	0.492	3.007	4233
School‐grade S.D. in SES (time‐varying)	0.862	0.127	1.235	4233
Track (time‐varying)	%			
Pre‐vocational	52.99			2243
Mixed/intermediate	30.55			1293
Academic	16.47			697
Total	100			4233
Migration background				
No migration background	78.55			3325
From an OECD country	2.55			108
From a non‐OECD country	18.9			800
Total	100			4233

*Note:* Descriptives on student‐grade data.

### 3.3 Modelling Strategy

We estimate growth curve models, modelling trends over grades. We first investigate baseline trends in civic and democratic engagement across the four secondary school grades 7–10, and furthermore tested for the covariance between the between‐student intercept variance and between‐student slope variance. With this exercise, we can examine whether the growth curve is statistically dependent on the initial level of engagement. We also test for non‐linear trends.

Then, we study the role of tracking for the development in civic and democratic engagement in three different ways. First, we study trends across grades between the three main tracks found in the Dutch system. We study the relation with track position in a dynamic way; allowing students to move across tracks, for instance from a mixed track in the first year(s) to an academic or pre‐vocational track in the later year(s). Equation (1) summarises this model, with subscripts *i* for individuals, *t* for grade, and *s* for school. Our model separates the socioeconomic gradient in a within‐school*grade (*SES_c*) and a between‐school*grade component (mSES) (Bell et al. 2019).

(1) $$\begin{array}{ll} y_{its}= & \alpha +\beta_{0}{Grade}_{its}+\beta_{1}{Track}_{its}+\beta_{2}{Grade}_{its}\times {Track}_{its}+\beta_{3}{SES\_c}_{its\;}+\beta_{4}{mSES}_{ts\;}+\beta_{5}{Migration}_{is}+\zeta_{s}+\xi_{is}^{1}\\ & +\xi_{is}^{2}{Grade}_{its}+\xi_{is}^{3}{Track}_{its}+\varepsilon_{its} \end{array}$$

Second, it is examined whether the impact of track depends on the extent to which schools bring students of different tracks together. This is done by creating a variable indicating the proportion of students in the first year of secondary school that is in the mixed track. This could vary from 0 in single‐track schools, to 1 for schools that only have students in mixed tracks (note that the maximum in our data is 0.97, Table 1). Equation (2) summarises this model, adding the three‐way interaction between grade, track, and the proportion of students who were in a mixed track in seventh grade. The model enables us to trace track differences as they emerge across schools that intentionally bring together students of different tracks and schools that intentionally only offer one track.

(2) $$\begin{array}{ll} y_{its}= & \alpha +\beta_{0}{Grade}_{its}+\beta_{1}{Track}_{its}+\beta_{2}{Grade}_{its}\times {Track}_{its}+\beta_{3}{Grade}_{its}\times {Pmixed}_{s}+\beta_{4}{Track}_{its}\times {Pmixed}_{s}\\ & +\beta_{5}{Grade}_{its}\times {Track}_{its}\times {Pmixed}_{s}+\beta_{6}{SES\_c}_{its\;}+\beta_{7}{mSES}_{ts\;}+\beta_{8}{Migration}_{is}+\zeta_{s}+\xi_{is}^{1}+\xi_{is}^{2}{Grade}_{its}\\ & +\xi_{is}^{3}{Track}_{its}+\varepsilon_{its} \end{array}$$

Third, in a similar vein we study how track differences vary across school compositions, both in terms of average SES (*mSES*) and the dispersion in socioeconomic status (*sdSES*) at the level of school*grade, see Equation (3).

(3) $$\begin{array}{ll} y_{its}= & \alpha +\beta_{0}{Grade}_{its}+\beta_{1}{Track}_{its}+\beta_{2}{Grade}_{its}\times {Track}_{its}+\beta_{3}{Grade}_{its}\times {mSES}_{ts}+\beta_{4}{Grade}_{its}\times {sdSES}_{ts}\\ & +\beta_{5}{Track}_{its}\times {mSES}_{ts}+\beta_{6}{Track}_{its}\times {sdSES}_{ts}+\beta_{7}{Grade}_{its}\times {Track}_{its}\times {mSES}_{ts}+\beta_{8}{Grade}_{its}\times {Track}_{its}\\ & \times {sdSES}_{ts\;}+\beta_{9}{SES_{c}}_{its\;}+\beta_{10}{mSES}_{ts\;}+\beta_{11}{Migration}_{is}+\zeta_{s}+\xi_{is}^{1}+\xi_{is}^{2}{Grade}_{its}+\xi_{is}^{3}{Track}_{its}+\varepsilon_{its} \end{array}$$

As a robustness check we also study more explicitly the transitions between two adjacent grades, and their relation to the difference score in the dependent variables. We distinguish three such transitions: (1) staying in the same track, (2) moving up, or (3) moving down. These transitions, and the difference score in the dependent variables, are calculated for each transition (grade 7–8, 8–9, 9–10). Pooling the (maximally) three transitions per individual, the difference score in the dependent variable is regressed on upward or downward moves between tracks in the system (relative to staying on track). Because we have multiple difference scores and transitions per individual, we add individual fixed effects (see Equation (4), now with $\zeta_{s}$ as student fixed effect). This model thus estimates how, within individuals, each transition is associated with a growth or decline in civic and democratic engagement.

(4) $$y_{it}-y_{it-1}=\alpha +\beta_{1}{Upward}_{t-1\to t}+\beta_{2}{Downward}_{t-1\to t}+\zeta_{s}+\varepsilon_{it}$$

## 4 Results

### 4.1 Growth Curve Models

The results of the basic growth curve models are displayed in Table 2. The table shows that the there is no overall growth over grades in the level of institutional trust or societal interest, but there is a growth in the intention to go to vote as adults and the level of political knowledge. There is significant between‐school variance in all four outcomes. There is also variance in the intercept and in the slope over grades between students, although in the case of the intercept in political knowledge not at a conventional statistical significance level. Importantly, for three of the four outcome variables there is an equalising tendency during the school years, indicated by a negative covariance between the intercept and the slope. This covariance indicates that students with a lower baseline level of engagement tend to have steeper growth curves.

**TABLE 2:** Growth curve models without covariates.

	Institutional trust		Societal interest		Voting intention		Political knowledge	
	est.	se	est.	se	est.	se	est.	se
Fixed part								
Grade	−0.014	0.012	−0.012	0.011	**0.054**	0.011	**0.157**	0.037
Constant	0.002	0.050	0.024	0.051	**2.007**	0.052	**3.368**	0.165
Random part								
Between‐school variance	**0.049**	0.016	**0.059**	0.018	**0.065**	0.019	**0.591**	0.157
Between‐student variance (intercept)	**0.474**	0.056	**0.348**	0.047	**0.435**	0.043	0.310	0.724
Between‐student variance of grade (slope)	**0.036**	0.008	**0.024**	0.007	**0.039**	0.006	**0.121**	0.083
Covariance intercept ‐ slope	**−0.081**	0.020	**−0.039**	0.016	**−0.090**	0.015	−0.085	0.237
Residual variance	**0.427**	0.015	**0.403**	0.014	**0.277**	0.010	**1.761**	0.105

*Note:* Growth curve models. For voting intention, the fit would improve with a curvilinear trend (based on Wald test).

*Source:* Dutch Adolescent Panel on Democratic Values (DAPDV). In **bold**: coefficients minimum twice the standard error.

To study variation between tracks and schools, we first estimate fit statistics of various models. Table 3 displays the results. Model one only adds main effects, of all individual‐level variables including track. Model 2 adds an interaction effect between track and grade, testing for track differences in the growth curve. Models 3 and 4 complicate this model further, by testing for differences in growth curves between tracks to vary across different types of schools. Model 3 does this by looking at the proportion of 7th‐grade students in mixed tracks, and Model 4 by looking at the school*grade composition in terms of socioeconomic status (its mean and its standard deviation). Note that the school composition is measured for each combination of school and grade separately. We report both the Baysian Information Criterion (BIC), of which lower values indicate better fit, and Wald statistics for the Chi‐square test whether the −2 log likelihood of a model improves relative to another (nested) model.

**TABLE 3:** Fit statistics of modelling trends across tracks.

	Institutional trust			Societal interest			Voting intention			Political knowledge		
	(*N* = 3605)			(*N* = 3768)			(*N* = 3599)			(*N* = 2498)		
	Wald test	Against model	BIC	Wald test	Against model	BIC	Wald test	Against model	BIC	Wald test	Against model	BIC
Model 1: Main effects (df = 13)			8855.6			8913.1			7573.3			9320.5
Model 2: M1 plus grade*track (df = 15)	10.09 (2)**	1	8861.9	1.79 (2)	1	8927.7	1.12 (2)	1	7588.6	4.32 (2)	1	9331.8
Model 3: M2 plus grade*track*proportion in mixed track (df = 21)	13.76 (8)^a^	1	8907.4	7.37 (8)	1	8971.6	15.48 (8)^a^	1	7623.3	21.57 (8)**	1	9361.5
	3.66 (6)	2		5.58 (6)	2		14.36 (6)*	2		17.25 (6)**	2	
Model 4: M2 plus grade*track*mSES and grade*track*sdSES (df = 26)	24.34 (13)*	1	8937.7	24.12 (13)*	1	8996.0	21.92 (13)^a^	1	7657.8	24.70 (13)*	1	9397.5
	14.25 (11)	2		22.32 (11)*	2		20.80 (11)*	2		20.39 (11)*	2	

*Note:* Models always include gender, Mundlak SES, and migration background. *N* of the student‐grade data.

^a^ *p* < 0.10.

^*^ *p* < 0.05.

^**^ *p* < 0.01.

Wald statistics reported in Table 3 show that for institutional trust, there are growth curve differences across tracks; for some tracks the growth over the grades is stronger than for other tracks. The track differences in growth curves do furthermore slightly vary across types of schools in terms of the proportion of mixed classes (at 90% confidence interval). There is a fit improvement in model 4 that allows for growth curves by track to vary across school SES composition.

For societal interest, there are no varying trends across tracks. There is some evidence that track differences are different across schools based on socioeconomic composition.

With regard to voting intention and political knowledge the results are quite different. Growth curves across tracks vary depending on the proportion of students in a mixed track. Moreover, also the track‐dependent growth curves vary across school SES compositions.

When we examine BIC statistics, that summarise the whole model fit, none of the models improved on the main effects model, suggesting that above‐mentioned fit improvements are modest if set off against the number of degrees of freedom.

**FIGURE 1:**
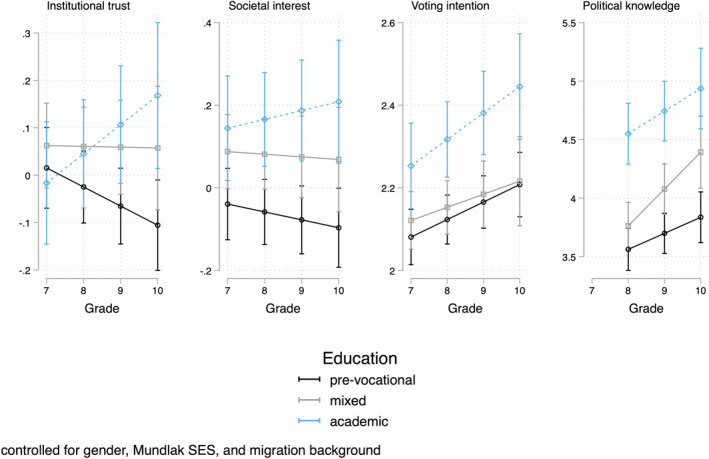
Marginal effects plots of growth curves by track (Model 2 of Table 3).

The results of these models are shown in the form of predicted outcomes on the variables using marginal effect plots. Figure 1 shows the results of model 2. With regard to institutional trust and societal interest, there is a pattern of divergence across tracks over the grades, with prevocational students show a downward trend and academic students an upward trend. The effect size is not negligible: the growth in the gap between academic and pre‐vocational students on institutional trust increases with about 0.2 standard deviations (with the standard deviation in institutional trust being 0.89, Table 1). Voting intention and political knowledge grow over the grades, with most growth among mixed/intermediate students for political knowledge. The overall trend could very well reflect that voting intention grows with age (Diemer and Li 2011). Note that track is, intentionally, measured in a time‐varying way, so the results can be due to unobserved selection in who moves from the mixed/intermediate track to the prevocational track or to the academic track.

**FIGURE 2:**
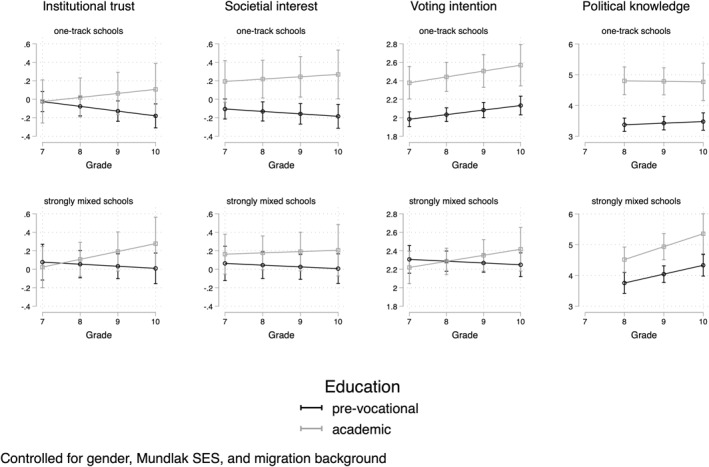
Growth curves in one‐track and strongly mixed schools.

Then, Figure 2 shows the results for Model 3, in which the track differences vary across schools with varying proportions of students in the mixed/intermediate track. Note that here only the effects are shown for students in the academic and pre‐vocational track, as they can move from a broad school with mixed‐track classes into the unitary track.^2^ Figure 2 shows trends across grades for students in the pre‐vocational and academic tracks, in single‐track schools relative to schools with a large proportion (80%) of seventh‐graders in the mixed track. If we compare vertically, we see that strongly mixed schools have, overall, smaller gaps between pre‐vocational and academic students than one‐track schools on all outcomes except institutional trust. The gaps between the tracks are small and confidence intervals fully overlap in mixed schools, but gaps are larger without overlapping confidence intervals in the single‐track schools. However, it can also be seen that most of the variation between the tracks in single‐track schools already exists at the start of seventh grade, and hence is more likely affected by processes of selection into the schools than by the resources or recruitment networks found in the different schools. Focusing on voting intention and political knowledge, because Table 3 showed that the inclusion of this school characteristic to the model improved the fit, we see voting intention to grow slightly more in one‐track schools relative to mixed schools, although the coefficients have large confidence intervals. With political knowledge, the growth is slightly stronger in mixed schools, although with a slightly steeper slope of the academic track.

**FIGURE 3:**
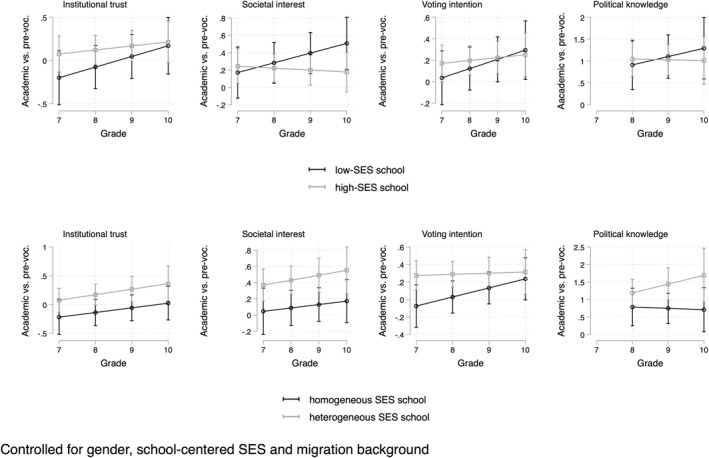
Growth curves by average SES and SES heterogeneity in schools.

Lastly, Figure 3 shows the results of model 4, allowing track differences to vary across school compositions in terms of socioeconomic status. The graphs above show results by average socioeconomic status, and the graphs below show the results by the standard deviation in socioeconomic status. On the vertical axis we now display the gap between academic and pre‐vocational education. Figure 3 shows that the track differences across the grades are not strongly related to the SES composition. All estimates are rather imprecise, and have large confidence intervals. Nevertheless, some interesting patterns are worth mentioning. The track gap seems to be slightly increasing in low‐SES schools, at least more than in high‐SES schools. When it comes to SES heterogeneity (standard deviation), the overall pattern is that the track gap is larger in heterogeneous schools.

### 4.2 Transition Models

**FIGURE 4:**
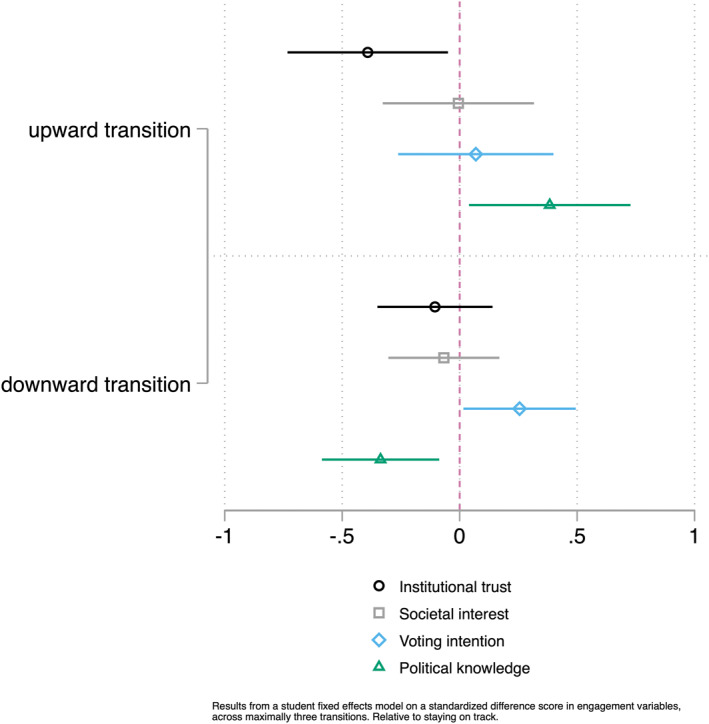
Results from the transition model.

As a robustness check, we study the temporal nature of the transitions and their possible related changes in civic and democratic engagement. Figure 4 shows the results of the transition model, focusing on upward and downward moves within individuals, over maximally three transitions per individual. The figure shows that an upward transition is associated with a decline in institutional trust of about 0.4 of a standard deviation, and a growth in political knowledge (also around 0.4 of a standard deviation). A downward transition is associated with a rise in voting intention (0.25 SD) and a decline in political knowledge (around 0.3 SD). So, when specifically addressing within‐individual moves through the educational system, there is evidence that educational stratification in political knowledge increases.

## 5 Conclusions and Discussion

This paper used newly collected panel data among secondary school students in the Netherlands, the Dutch Adolescent Panel on Democratic Values (DAPDV), to study the development of civic and democratic engagement of students in different tracks. It is important to understand possible track differences, because schooling can equalise engagement with democracy and promote social cohesion but only if tracks do not further differentiate initial inequalities. Track differences have been studied in a number of earlier studies, but the results are mixed. Sometimes track effects are found, sometimes not, although the literature is rather scattered and inconclusive. It was our aim to study track differences in different school contexts, in particular with regard to bringing students of various tracks and various socioeconomic backgrounds together in one school setting. Moreover, our panel study enabled us to study trends across tracks over 4 years of schooling, and to examine various indicators of civic and democratic engagement.

Our results indicated that for some of the outcomes we studied, in particular those that are more directly connected to politics, a growth pattern was observed across the 4 years. Students become more inclined to go to vote when they turn into adulthood, and acquire more political knowledge during secondary school. Moreover, we found that students with a lower initial level of civic and democratic engagement had steeper growth curves, suggesting that there is a tendency toward equalisation of democratic and civic engagement. An alternative explanation, namely that there are ceiling effects in the measurement of engagement making it harder to grow from higher initial scores, can however not be ruled out.

Track differences in civic and democratic engagement are observed. With regard to institutional trust we saw track differences to increase, with students in the academic track showing greater growth. But overall track differences are rather stable over the school career. In other words, the differences we observe between tracks already existed for a large part at the start of secondary school, suggesting that the (prevocational, intermediate, or academic) track that students enrol does not have a causal effect on democratic and civic engagement. Models that focused explicitly on the upward and downward transitions within individuals found that upward transitions came with a rise in political knowledge, and downward transitions with a decline in political knowledge.

It is worthwhile to discuss our findings more explicitly to two studies that employed a rather similar design as ours. First, Eckstein et al. (2012) also examined trends between grades 7 and 11, using similar growth curve models, using data from the German state of Thuringia. They find stronger growth in the intention to take part in politics among students in the academic track than in other tracks. Importantly, they used a scale for various political activities, such as being member of an environmental group or a student council, donating money, and taking part in demonstrations. Our measure of intention to political participation was more narrowly focusing on the intention to vote at elections, which could possibly explain the discrepancies. Another explanation for the different findings could be that there are educational institutional differences between Thuringia and the Netherlands, with students in Thuringia selected after grade 4 and in the Netherlands after grade 6.

Second, Hoskins and Janmaat (2016) examined differences in political participation across tracks in a pre‐post design, using panel data from the United Kingdom. Their results showed that track differences in having voted at the national election at age 19/20 were significant, but reduced after controlling for pre‐track intentions to vote. Possibly some of their track effects remained because the first measurement was done before students were allocated to a track. In our study, while the first data collection happened in October of the first year in high school, students had already been informed about their educational position, hence some track effects may have already been established. We would argue that such differences are not likely to be attributable to experiences in the tracks themselves, as educational and network processes are unlikely to have produced such differences within one or 2 months of schooling. Moreover, the timing of data collection vis‐à‐vis the pre‐track measurement does not explain the discrepancy with Germany mentioned above, as the track effects are assessed a few years after the tracking started.

The strong evidence of selection effects supports the “education‐as‐proxy” explanation for education gaps in political outcomes, stating that education merely proxies earlier life experiences. Given that the education‐as‐proxy argument gets supported in a study that starts to collect educational data around the age of 12 illustrates that confounding factors are already important at stages when educational differences are just about to start. However, the fact that there is growth over the years (at least with regard to the politically oriented outcomes, and with upward moves in the system) is compatible with the assumption that education can contribute to the development of civic and democratic engagement, and that upward transitions may lead to more engagement growth than other transitions. Hence, secondary education—followed by all youngsters in society—can have a causal effect on engagement. From this perspective, more insight is needed into how civic education takes shape in different tracks and student engagement as outcome of various curriculum designs.

Looking more in detail into track differences in different types of schools, it appears that track differences are stronger in single‐track schools than in schools with broader mixed classes at the start of secondary school. These differences were, again, already present in the first observed year, suggesting that selection into schools based on unobserved correlates of engagement account for much of these between‐school differences. It should be noted that socioeconomic background was controlled for, both within and between school*grade combinations, so socioeconomic selection seems not to be the crucial factor here. But other candidates are academic performance or intelligence, factors that matter for the type of schools students enrol in, and which are likely predictors of civic and political orientation (Schoon et al. 2010). With regard to socioeconomic composition, there was little relationship with the size of the track gap. Some indications exist that track differences are a bit larger in heterogeneous schools with regard to socioeconomic status than in homogeneous schools.

We posited two theories of how school context may matter for track differences in engagement. The first theory holds that schools that bring together students of various tracks and socioeconomic backgrounds may form environments in which between‐group differences are less pronounced, and develop to become more equal over the years (*integration hypothesis*). We found that track differences were indeed smaller in broader schools, which is suggestive of a less diversified political environment. Yet, such broader schools did not diminish track differences over time. The second theory, summarised in the *polarisation hypothesis*, holds that track differences get more pronounced if students are brought together. Difference between tracks may become more meaningful in a context where students of various tracks are in the same environment, and identification with one's own group could strengthen between‐group differences in engagement. Two, at best suggestive, findings in line with this hypothesis are, first, that track differences in political knowledge increased especially in mixed schools, and second, that track differences were larger in schools with more heterogeneity with regard to socioeconomic status. But overall there is not clear support for either the integration or the polarisation hypothesis. We cannot rule out the possibility that both mechanisms are at work, resulting in a null effect of type of school.

Some caveats to our study are worth mentioning. First, while we classify schools as mixed based on the extent to which students of different tracks are brought together in the first year, we do not know how schools arrange tracks across the full duration of secondary schools. Some schools organise their tracks into separate buildings after 2 years, other schools keep students of different tracks together in the same environment. Such choices, unobserved in our design, are potentially important for integration or polarisation. Second, we do not know how schools organise civic and citizenship education. It is possible that more actively engaged schools affect differences between students (Mennes et al. 2023), in correlated or uncorrelated ways to the institutionalised track differentiation.

What do these results tell us about the relevance of tracking for civic and democratic engagement? This question has three components, relating to the individual track trajectory, the school the students attend, and the educational system in the society. As regards individual track, track mostly matters through selection. Growth rates across years were similar across tracks, although some suggestive findings of divergence were found. As regards the school, bringing students together from different tracks is associated with reduced democratic gaps between tracks. In that sense, broad schools create environments where different recruitment networks are established. Whether those broad school environments are (causally) effective is another matter; there is not much evidence in our study that engagement gaps between tracks change in relation to the integrative character of schools. Then, at the system level the question whether a tracking system enhances democratic inequalities in a society, as has been argued before (Janmaat and Mons 2011; Van de Werfhorst 2017; Witschge and Van de Werfhorst 2020). Of course the current findings cannot directly inform us about cross‐national differences. The counterfactual of a tracked educational system is a comprehensive educational system, and we have not observed such a system. It could be that tracking systems are detrimental to civic and democratic engagement relative to comprehensive systems, but that between‐track trends within a tracked system are stable.

## Ethics Statement

We obtained ethical approval from the Amsterdam Institute for Social Science Research of the University of Amsterdam (ERB number 2018‐AISSR‐9324). An earlier version of this paper has been presented at the 2024 conference of the European Consortium for Sociological Research, in Barcelona.

## Conflicts of Interest

The authors declare no conflicts of interest.

## Data Availability

The data that support the findings of this study are available on request from the corresponding author. The data are not publicly available due to privacy or ethical restrictions.

## Endnotes

^1^
Results were very similar if we had included all students, independent of their presence in the first wave and the number of waves present.

^2^
Students in the mixed/intermediate track in later grades are usually situated in the intermediate (havo) track, rather than in a properly mixed track, as mixed tracks mostly exist in the first one or two grades of secondary school. Hence, the effect of a mixed/intermediate track in later grades would result from intermediate track‐students coming from either mixed or unitary intermediate school in previous grades. With the pre‐vocational and academic track it is much less likely that they originate from a unitary intermediate school, unless they went from a unitary intermediate school to a unitary pre‐vocational or academic school. Thus, if they originate from a school with many students in the mixed/intermediate category in seventh grade, it is highly likely that they do originate from a school with a mixed track.
